# Effects of Upper-Body Plyometric Training on Physical Fitness in Healthy Youth and Young Adult Participants: A Systematic Review with Meta-Analysis

**DOI:** 10.1186/s40798-023-00631-2

**Published:** 2023-10-13

**Authors:** Exal Garcia-Carrillo, Rodrigo Ramirez-Campillo, Rohit K. Thapa, José Afonso, Urs Granacher, Mikel Izquierdo

**Affiliations:** 1 Navarrabiomed-Universidad Pública de Navarra (UPNA), Complejo Hospitalario de Navarra (CHN), Instituto de Investigación Sanitaria de Navarra (IdiSNA), 31008 Pamplona, Navarra Spain; 2 Exercise and Rehabilitation Sciences Institute, Faculty of Rehabilitation Sciences, School of Physical Therapy, Universidad Andres Bello, 7591538 Santiago, Chile; 3 Symbiosis School of Sports Sciences, Symbiosis International (Deemed University), Pune, 412115 India; 4 Faculty of Sport, Centre of Research, Education, Innovation, and Intervention in Sport (CIFI2D), University of Porto, 4200-450 Porto, Portugal; 5 Department of Sport and Sport Science, Exercise and Human Movement Science, University of Freiburg, Freiburg, Germany; 6 Department of Health Sciences, Public University of Navarra, Av. De Barañain s/n, 31008 Pamplona, Navarra Spain

**Keywords:** Plyometric exercise, Muscle strength, Musculoskeletal physiological phenomena, Human physical conditioning, Resistance training, Athletic performance, Sports medicine

## Abstract

**Background:**

Upper-body plyometric training (UBPT) is a commonly used training method, yet its effects on physical fitness are inconsistent and there is a lack of comprehensive reviews on the topic.

**Objective:**

To examine the effects of UBPT on physical fitness in healthy youth and young adult participants compared to active, specific-active, and passive controls.

**Methods:**

This systematic review followed PRISMA 2020 guidelines and utilized the PICOS framework. PubMed, WOS, and SCOPUS were searched. Studies were assessed for eligibility using the PICOS framework. The effects of UBPT on upper-body physical fitness were assessed, including maximal strength, medicine ball throw performance, sport-specific throwing performance, and upper limb muscle volume. The risk of bias was evaluated using the PEDro scale. Means and standard deviations were used to calculate effect sizes, and the *I*^2^ statistic was used to assess heterogeneity. Publication bias was assessed using the extended Egger's test. Certainty of evidence was rated using the GRADE scale. Additional analyses included sensitivity analyses and adverse effects.

**Results:**

Thirty-five studies were included in the systematic review and 30 studies in meta-analyses, involving 1412 male and female participants from various sport-fitness backgrounds. Training duration ranged from 4 to 16 weeks. Compared to controls, UBPT improved maximal strength (small ES = 0.39 95% CI = 0.15–0.63, *p* = 0.002, *I*^2^ = 29.7%), medicine ball throw performance (moderate ES = 0.64, 95% CI = 0.43–0.85, *p* < 0.001, *I*^2^ = 46.3%), sport-specific throwing performance (small ES = 0.55, 95% CI = 0.25–0.86, *p* < 0.001, *I*^2^ = 36.8%), and upper limbs muscle volume (moderate ES = 0.64, 95% CI = 0.20–1.08, *p* = 0.005, *I*^2^ = 0.0%). The GRADE analyses provided low or very low certainty for the recommendation of UBPT for improving physical fitness in healthy participants. One study reported one participant with an injury due to UBPT. The other 34 included studies provided no report measure for adverse effects linked to UBPT.

**Conclusions:**

UBPT interventions may enhance physical fitness in healthy youth and young adult individuals compared to control conditions. However, the certainty of evidence for these recommendations is low or very low. Further research is needed to establish the optimal dose of UBPT and to determine its effect on female participants and its transfer to other upper-body dominated sports.

**Supplementary Information:**

The online version contains supplementary material available at 10.1186/s40798-023-00631-2.

## Key Points

- Upper-body plyometric training can be effective at improving maximal strength, medicine ball throwing performance, sport-specific throwing performance, and muscle volume in healthy youth and young adult participants.
- An effective dose of a progressive overload UBPT programme may involve: a minimal duration of 4 weeks, 2–4 sessions/week, 1–6 exercises/session, 1–10 sets per exercise, a mean of ~ 12 repetitions/set (range 3–30; lower range usually with maximal effort-intensity), and an inter-set and inter-session rest of 15–240 s and 48–96 h, respectively.
- The findings of this meta-analysis were derived from 30 articles with low risk of bias (good methodological quality), low study heterogeneity, and low to very low certainty of evidence (GRADE), encompassing a total of 1,412 participants ranging from 7.3 to 27.2 years of age.

**Supplementary Information:**

The online version contains supplementary material available at 10.1186/s40798-023-00631-2.

## Introduction

Upper-body strength-related measures (e.g., maximal strength; rate of force development), and anthropometric characteristics, have been shown to differentiate sub-elite and elite athletes [1, 2]. Research has also demonstrated the positive relationship between upper-body power measures, such as bench press throws and upper-body Wingate test [3, 4], and strength measures, such as the bench pull, bench press, and pulldown test [5–8], with athletic performance in various sports, including golf, rugby, handcycling, (sprint) swimming, double poling, and (sprint) kayaking. Furthermore, markers of upper-body strength, such as one repetition maximum (1RM) for the bench press and bench row or handgrip strength, have proven to be useful for identifying talented athletes in kayaking, cross-country skiing, and tennis [9–11]. In addition to its relevance to performance, upper-body strength-related measures can impact training dosage, as weaker individuals may require longer rest intervals between sets [12, 13]. Moreover, upper-body strength, particularly handgrip strength, is a prognostic indicator of morbidity and all-cause mortality [14–16], and can predict fall severity in older adults [17, 18]. Therefore, upper-body strength-related measures are important from both performance and health-related perspectives. The development of safe, effective, and convenient training methods that can improve these outcomes is necessary.

Various methods of upper-body resistance training are commonly used by strength and conditioning specialists, coaches, and athletes [19]. In this context, upper-body plyometric training (UBPT) offers some advantages over other training approaches. UBPT is effective in improving measures of power [20] and strength [21] through exercises that maximize the storage and use of elastic energy through the muscle stretch–shortening cycle, typically with a brief transition period between the stretching and shortening phases of muscle actions [22–24]. UBPT exercises, such as bench press throws and medicine ball throws, can be added to heavy resistance training, such as the bench press and dumbbell shoulder press, to improve power-related performance, such as medicine ball throwing speed [25], and maximal upper-body strength such as 1RM bench press [26]. This can be particularly relevant in sports where powerful upper-body actions are more common, such as golf, rugby, handcycling, and baseball. Therefore, UBPT is considered a time-efficient training method [27, 28]. Additionally, UBPT is well-suited for sports, such as handball and volleyball, that require powerful upper-body actions as it mimics the motor patterns demanded by these sports more closely than non-plyometric strength training methods.

Research studies have demonstrated that plyometric training can significantly improve sport-specific performance in sports like soccer (e.g., kicking velocity) [29–31] and swimming [32]. However, the transferability of these findings into practical settings has been limited due to the small sample sizes of most published studies [33]. Studies investigating UBPT often have small sample sizes (e.g., *n* < 13) [34, 35], which can be a drawback in the sport science literature [36]. To overcome this issue, systematic reviews with meta-analyses can be useful in evaluating the results of comparable studies and helping practitioners make evidence-based decisions [37, 38]. While previous systematic reviews on plyometrics have mainly focused on lower body training [33, 39], some conflicting findings have been reported regarding the effectiveness of UBPT in enhancing physical performance parameters like strength, power, and throwing speed [40, 41].

A prior systematic review, which included a database search up to August 2017 [42] assessed the impact of UBPT on strength, ball throwing speed, distance and power in healthy individuals, including only six randomized controlled trials. Given the rapid growth in the field of plyometric training research in the recent years, with the rate of yearly publications increasing almost 16-fold between 1980–1999 and 2000–2019 [33], it is crucial to re-evaluate the scientific literature. In rapidly emerging research fields, 25–50% of systematic reviews may be obsolete within 2–5 years [43]. Therefore, the primary objective of this systematic review with meta-analysis was to examine the effects of UBPT, compared with active/passive controls, on the physical fitness of healthy youth and young adult participants.

## Methods

### Registration

The systematic review with meta-analysis protocol was registered on the Open Science Framework (OSF) platform, in January 13, 2023 (registration code: XQ25T; registration https://doi.org/10.17605/OSF.IO/XQ25T).

### Procedures

The procedures were in line with the Preferred Reporting Items for Systematic Reviews and Meta-Analyses (PRISMA) 2020 guidelines [44] and the Cochrane Handbook for Systematic Reviews of Interventions [45].

### Literature Search: Administration and Update

A systematic literature search was performed without date restriction, and updated up to February 2023, in the electronic databases PubMed, Web of Science, and SCOPUS, using the Boolean operators AND/OR, in combinations with the keywords “plyometric”, “explosive”, “ballistic”, “upper body”, and “upper limb”. An exemplified combination and the search strategy (code line) for each database is described in the Additional file 1: Table S1. One author (EGC) conducted the initial search and removed duplicates. Two authors (EGC and RKT) independently screened the titles, abstracts, and full-texts of the retrieved studies. The search results were then analysed according to the eligibility criteria (Table 1). A third author (RRC) resolved potential disagreements between EGC and RKT. Although not considered in the original protocol, to expand the scope of our search and better abide by AMSTAR 2 guidelines [46], we contacted two experts in the field of UBPT (https://expertscape.com/ex/plyometric+exercise), sharing with them our inclusion/exclusion criteria and a detailed table of articles (i.e., authors, publication year, identifier, title, and journal) that we have identified as potential candidates for inclusion and asked if they were aware of any additional studies that could be added to the review.

**Table 1:** Eligibility criteria

Category	Inclusion criteria	Exclusion criteria
Participants	Healthy individuals, without restrictions on their fitness level, sex, or age	Studies that included participants with health issues (e.g., upper-body injuries, recent surgery) that prevent them from either performing exercise or doing it at maximum intensity
Interventions	UBPT programs with a minimal duration of ≥ 3 weeks [33, 39] which commonly utilize a pre-stretch or countermovement stressing the stretch–shortening cycle	Interventions not including UBPT; or those including UBPT but in combination with other type of exercises with an augmented representation (i.e., > 50% of total number of exercises derived from non-UBPT drills) so that the independent effect of UBPT was precluded)
Comparators	Active (i.e., individuals regularly involved in training schedules), specific-active, or non-active control groups	Absence of a control group
Outcomes	Studies that reported health- (e.g., muscle mass) and skill-related (e.g., power; joint angle-velocity during shot-put) physical fitness outcomes before and after UBPT	Studies that did not report baseline and/or follow-up data
Study design	Randomized controlled and non-randomized controlled trials	Studies including only one group, case studies, observational studies

UBPT: upper-body plyometric training

### Inclusion and Exclusion Criteria

Original, peer-reviewed, full-text studies were selected, and included/excluded using a PICOS (participants, interventions, comparators, outcomes, and study design) framework [47] (Table 1). Additional exclusion criteria are provided as Additional file 1: Table S2. As 99.6% of the plyometric training literature is published in English [39], and due to limited resources, only articles written in English, French, German, Italian, Portuguese, and Spanish (i.e., authors' native languages), were considered for inclusion.

### Data Extraction

The effects of UBPT on upper-body physical performance measures were assessed, compared to active (e.g., athletes in standard training), specific-active (e.g., a group performing high-load resistance training) and/or non-active controls. Performance measures included (but not limited to) different specific tests (e.g., bench press, medicine ball throw) and indices (e.g., kg; power [W]). Measures like the powerful medicine ball throw (intra-class correlation coefficient = 0.93–0.99) and the bench press throw (intra-class correlation coefficient = 0.94–0.85; coefficient of variation = 2.48%) have shown excellent reliability among competitive or physically active individuals [48, 49], thus favouring meta-analysis consistency [47].

Pre- and post-intervention means and standard deviations of the dependent variables were extracted from the included studies using Microsoft Excel (Microsoft Corporation, Redmond, WA, USA). For studies reporting values other than means and standard deviation (e.g., median, standard error), conversion was applied [50–52] or appropriate statistical software was used for different data formats (Comprehensive Meta-Analysis Software, Version 2, Biostat, Englewood, NJ, USA). When the required data were not clearly or completely reported, the authors of the respective studies were contacted for clarification purposes. If no response was obtained from the authors or the authors did not provide the requested data, the study outcome was excluded from further analysis. When needed, a validated [53] software (WebPlotDigitizer, version 4.5; https://apps.automeris.io/wpd/) was used to obtain numerical data from articles that reported results in figures. One author (EGC) performed data extraction, a second author (RKT) provided confirmation, and a third author (RRC) helped to resolve any discrepancies.

### Risk of Bias of the Included Studies

Included studies were assessed with a valid and reliable tool (i.e., PEDro scale) [54–56], probably the most frequently used in the plyometric training literature [33, 57, 58]. Despite being called a "methodological quality" scale, its items assess aspects of the likelihood that research will be biased. Therefore, it is helpful to compare meta-analyses, especially when they used different risk of bias assessment methodologies [59, 60]. Considering that it is not possible to satisfy all scale items in UBPT interventions [61], the overall risk of bias of studies was interpreted using the following convention [57, 61–63]: ≤ 3 points was considered as “poor” quality (i.e., high risk of bias), 4–5 points was considered as “moderate” quality, while 6–7 points and 8–10 points was considered as “good” and “excellent” quality, respectively. For practical purposes and given the nature of the research field, we considered studies with ≥ 6 points to have low risk of bias. Two authors (EGC and RKT) independently assessed risk of bias, and a third author (RRC) helped to resolve discrepancies.

### Summary Measures, Synthesis of Results, and Publication Bias

Meta-analyses can be conducted with as little as two studies [64]. However, meta-analyses were performed if ≥ 3 studies were available due to the reduced number of participants commonly included in plyometric training studies [65–67]. Meta-analysis was performed using the DerSimonian and Laird random-effects model [68, 69], with the means and standard deviations from pre and post values taken to compute effect sizes (ES, i.e., Hedges' *g*, presented with 95% confidence intervals [95% CIs]) for performance parameters in the UBPT compared to the control groups. Data were standardised using post-intervention standard deviation values. Calculated ES were interpreted as trivial (< 0.2), small (0.2–0.6), moderate (> 0.6–1.2), large (> 1.2–2.0), very large (> 2.0–4.0), and extremely large (> 4.0) [70]. A proportional division treatment to the control group *n* was applied in studies including multiple intervention groups and a single control group [71]. The *I*^2^ statistic was used to assess the impact of heterogeneity, with values of < 25%, 25–75%, and > 75% representing low, moderate, and high levels of heterogeneity, respectively [72]. The extended Egger’s test was used to assess risk of publication bias for continuous variables (≥ 10 studies per outcome) [73–75] and a sensitivity analysis was conducted with the trim and fill method for adjustments [76], with L0 as the default estimator for the number of missing studies [77]. All analyses were carried out using the Comprehensive Meta-Analysis Software (Version 2, Biostat, Englewood, NJ, USA). Statistical significance was set at *p* ≤ 0.05.

### Additional Analyses

#### Sensibility Analyses

The robustness of the summary estimates (e.g., *p*-value, ES, *I*^2^) for each outcome was analysed with each study deleted from the model (automated leave-one-out analysis).

#### Certainty of Evidence

Two authors (JA and RRC) rated the certainty of evidence (i.e., high; moderate; low; very low) using the Grading of Recommendations, Assessment, Development and Evaluation (GRADE) [78–80]. The evidence started at a high level of certainty (per outcome), but was downgraded based on the following criteria: (i) *Risk of bias in studies*: judgments were downgraded by one level if the median PEDro scores were moderate (< 6) or by two levels if they were poor (< 4); (ii) *Indirectness*: low risk of indirectness was attributed by default due to the specificity of populations, interventions, comparators and outcomes being guaranteed by the eligibility criteria; (iii) *Risk of publication bias*: downgraded by one level if there was suspected publication bias; (iv) *Inconsistency*: judgments were downgraded by one or two levels when the impact of statistical heterogeneity (*I*^2^) was moderate (≥ 25%) or high (> 75%); (v) *Imprecision*: one level of downgrading occurred whenever < 800 participants were available for a comparison [81] and/or if there was no clear direction of the effects. When both were observed, certainty was downgraded by two levels.

#### Adverse Effects

The potential adverse health effects, derived from the inadequate implementation of UBPT interventions, were qualitatively assessed.

## Results

### Study Selection

**Fig. 1:**
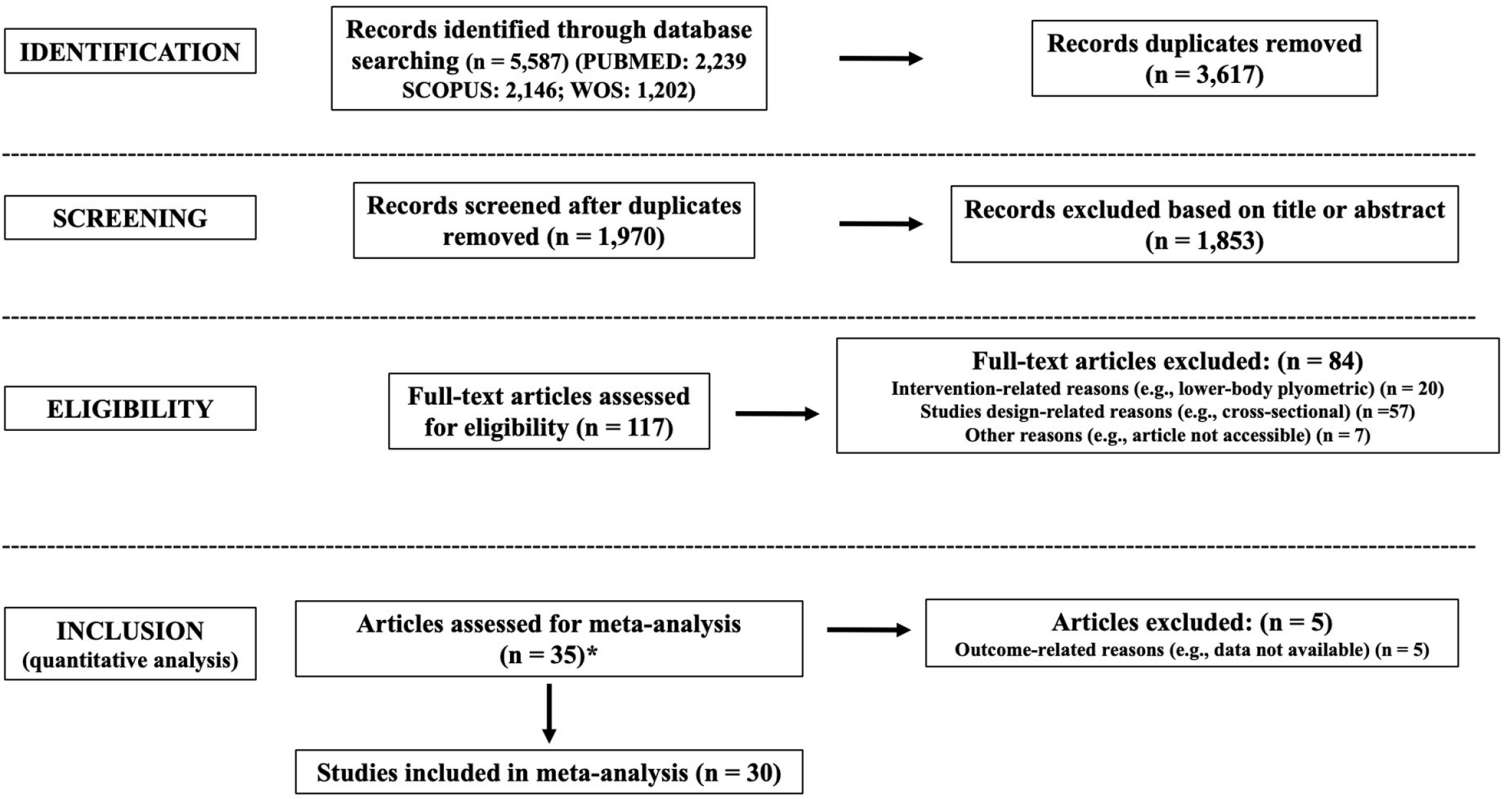
Flow diagram of the systematic search process. (*): denotes that 2 studies were identified through other sources (i.e., previous systematic review)

The search process in the databases identified 5587 records. Figure 1 provides a flow chart illustrating the study selection process.

Duplicate records were removed (n = 3617). After titles and abstracts were screened, 1853 records were removed and 117 full texts were evaluated. Thereafter, 35 studies were considered eligible for the systematic review, and 30 studies for the meta-analyses. The exclusion reasons from the meta-analysis for the five studies [20, 82–85] are indicated in Fig. 1. From the 30 studies eligible for meta-analyses 29 were written in English [21, 26, 28, 40, 41, 86–109] and one in Spanish [110].

### Risk of Bias of the Included Studies

According to the PEDro checklist results (Table 2), the median (i.e., non-parametric) score was 6.0 (low risk of bias—good quality), with nine studies attaining 4–5 points (some risk of bias—moderate quality), and 26 studies attaining 6 points (low risk of bias—good quality).

**Table 2:** Rating of studies according to the Physiotherapy Evidence Database (PEDro) scale

	1	2	3	4	5	6	7	8	9	10	11	Score^a^	Study quality
Aloui et al. [86]	1	1	0	1	0	0	0	1	1	1	1	6	Good
Alvarez et al. [87]	1	1	0	1	0	0	0	1	1	1	1	6	Good
Bouagina et al. [88]	1	1	0	1	0	0	0	0	1	1	1	5	Moderate
Carter et al. [89]	1	1	0	1	0	0	0	0	1	1	1	5	Moderate
Chelly et al. [90]	1	1	0	1	0	0	0	1	1	1	1	6	Good
Cuevas-Aburto et al. [91]	1	1	0	1	0	0	0	1	1	1	1	6	Good
Faigenbaum et al. [92]	1	1	0	1	0	0	0	1	1	1	1	6	Good
Fernandez-Fernandez et al. [93]	1	1	0	1	0	0	0	1	1	1	1	6	Good
Fernandez-Fernandez et al. [94]	1	0	0	1	0	0	0	0	1	1	1	4	Moderate
Haghighi et al. [95]	1	1	0	1	0	0	0	0	1	1	1	5	Moderate
Hammami et al. [96]	1	1	0	1	0	0	0	1	1	1	1	6	Good
Hasan et al. [82]	1	1	0	1	0	0	0	1	1	1	1	6	Good
Heiderscheit et al. [40]	1	1	0	1	0	0	0	1	1	1	1	6	Good
Ioannides et al. [97]	1	0	0	1	0	0	0	1	1	1	1	5	Moderate
Mangine et al. [26]	1	1	0	1	0	0	0	1	1	1	1	6	Good
Marta et al. [98]	1	1	0	1	0	0	0	1	1	1	1	6	Good
Martins et al. [99]	1	1	0	1	0	0	0	1	1	1	1	6	Good
Newton et al. [41]	1	1	0	1	0	0	0	1	1	1	1	6	Good
Pardos-Mainer et al. [110]	1	0	0	1	0	0	0	1	1	1	1	5	Moderate
Pereira et al. [20]	1	1	0	1	0	0	0	1	1	1	1	6	Good
Pienaar et al. [100]	1	1	0	1	0	0	0	1	1	1	1	6	Good
Santos et al. [28]	1	1	0	1	0	0	0	1	1	1	1	6	Good
Santos et al. [101]	1	1	0	1	0	0	0	1	1	1	1	6	Good
Schulte-Edelmann et al. [83]	1	1	0	1	0	0	0	1	1	1	1	6	Good
Singh Vishen et al. [84]	1	1	0	1	0	0	0	1	1	1	1	6	Good
Singla et al. [102]	1	1	0	1	0	0	0	1	1	1	1	6	Good
Sortwell et al. [103]	1	1	0	1	0	0	0	1	1	1	1	6	Good
Swanik et al. [85]	1	1	0	1	0	0	0	1	1	1	1	6	Good
Szymanski et al. [104]	1	1	0	1	0	0	0	1	1	1	1	6	Good
Thaqi et al. [105]	1	0	0	1	0	0	0	1	1	1	1	5	Moderate
Turgut et al. [106]	1	1	0	1	0	0	0	1	1	1	1	6	Good
Valades et al. [21]	1	0	0	1	0	0	0	1	1	1	1	5	Moderate
Vossen et al. [107]	1	1	0	1	0	0	0	1	1	1	1	6	Good
Wilson et al. [108]	1	1	0	1	0	0	0	1	1	1	1	6	Good
Young et al. [109]	1	0	0	1	0	0	0	1	1	1	1	5	Moderate

^a^From a possible maximal score of 10. A detailed explanation for each PEDro scale item can be accessed at https://www.pedro.org.au/english/downloads/pedro-scale; In brief: item 1, eligibility criteria were specified; item 2, participants were randomly allocated to groups; item 3, allocation was concealed; item 4, the groups were similar at baseline; item 5, there was blinding of all participants regarding the upper-body plyometric training programme being applied; item 6, there was blinding of all coaches responsible for the application of the upper-body plyometric training programme regarding its aim; item 7, there was blinding of all assessors involved in measurement of upper-body outcomes; item 8, measures of outcome variables were obtained from more than 85% of participants initially allocated to groups; item 9, all participants for whom upper-body outcomes were available received the treatment or control condition as allocated or, data were analysed by “intention to treat”; item 10, the results of between-group statistical comparisons were reported; and item 11, measures of variability for at least one upper-body outcome were provided

### Study Characteristics

The characteristics of the participants and the UBPT programs of the included studies are detailed in Table 3.

**Table 3:** Descriptive characteristics of participants and upper-body plyometric training interventions

Study	Rand	Sex	Age (y)	Body mass (kg)	Height (cm)	Sport	Fit	Fr	Weeks	UBPT exercises
Aloui et al. [86]	Yes	NR	17.7/18.1^a^	76.8/73.7	183.0/182.0	Handball	Mo-H	2	8	Plyometric standard push-up, plyometric diamond and wide arm push-up (all with elastic bands)
Alvarez et al. [87]	Yes	M	24.2/23.9	68.1/70.8	171.0/172.0	Golf	H	2	6	Horizontal BP + plyometric push-up, seated row machine + explosive pull-downs, seated barbell military press + plyometric push-up, triceps cable push-down + plyometric push-up
Bouagina et al. [88]	Yes	M	17.6/17.4	79.1/82.4	181.0/184.0	Handball	Mo	2	8	Arm/shoulder specific strength device
Carter et al. [89]	Yes	NR	19.7	90.7	183.0	Baseball	Mo	2	8	"Ballistic Six" (latex tubing external rotation, latex tubing 90/90 external rotation, overhead soccer throws with a 2.7 kg MB, 90/90 external rotation side-throw with a 0.9 kg MB, deceleration baseball throws with a 0.9 kg MB, and baseball throw with 0.9 kg
Chelly et al. [90]	Yes	M	17.1/17.2	80.1/78.0	181.0/177.0	Handball	Mo	2	8	Dynamic push-up
Cuevas-Aburto et al. [91]	Yes	M	22.6/24.3	80.3/81.1	177.0/176.0	Handball	N	2	8	BP throw at 40% of 1RM
Faigenbaum et al. [92]	Yes	M	13.4/13.6	61.5/58.6	164.0/166.0	Softball	N	2	6	MB overhead throw, MB chest pass, MB backwards throw, MB lunge chest pass, MB partner push pass (1–2 kg MB)
Fernandez-Fernandez et al. [93]	Yes	M	13.2/13.2	49.8/49.8	161.0/163.0	Tennis	Mo-H	3	6	MB chest pass, MB overhead throw, MB Ear throw, MB overhead slam, MB close-stance throw -2 kg MB-
Fernandez-Fernandez et al. [94]	No	M	12.5	44.2	156.0	Tennis	Mo	2	8	Push-up, push-up (clapping hands), MB chest throw, MB overhead throw, MB close stance throw, MB open-stance throw, MB two-hand overhead throw with rotation, MB overhead slam, (2 kg MB)
Haghighi et al. [95]	Yes	M	24.0	74.0	175.0	Table Tennis	Mo	3	8	MB throws on chest, MB throws on the right side, MB throws on the left side (3 kg MB)
Hammami et al. [96]	Yes	F	13.5/13.3	42.6/42.3	142.0/143.0	Handball	Mo	2	9	Dynamic push-up
Hasan et al. [82]	Yes	M	22.7/23.3	59.1/56.6	160.9/167.3	Basketball	N-Mo	2	6	MB double arm overhead throw, MB double arm chest pass, MB double arm side-to-side throw, MB double arm through leg throw (2.7–3.6 kg MB)
Heiderscheit et al. [40]	Yes	F	19/19.2	58.9/60.6	166.6/166.3	NA	L	2	8	Dominant arm weighted ball throws (1.36 and 1.82 kg)
Ioannides et al. [97]	No	Mix	17.6/17.0	65.1/67.1	172.0/177.0	Karate	Mo-H	2	6	MB seated throw (1 kg)
Mangine et al. [26]	Yes	M	21.4/20.1	82.7/81.0	176.8/180.8	RT	N	3	8	BP throw, MB chest pass, MB twist throw, MB sit-up throw
Marta et al. [98]	Yes	Mix	10.8	39.0	141.0	NA	N	2	8	MB Chest throw (1 kg), MB chest throw (3 kg), MB overhead throw (1 kg), MB overhead throw (3 kg)
Martins et al. [99]	Yes	Mix	17.6/17.6	NR	NR	NA	N	2	8	MB throws (1 kg and 3 kg)
Newton et al. [41]	Yes	M	18.6	73.7	179.0	Baseball	N-Mo	2	8	MB chest pass, MB overhead throw (3 kg)
Pardos-Mainer et al. [110]	No	M/F	16.3/12.6	70.9/53.6	181.4/161.8	Tennis	H	2	8	MB chest pass, MB overhead pass, MB closed position pass, clap push-up (girls = 2 kg MB; boys = 3 kg MB)
Pereira et al. [20]	Yes	F	14.0/13.8	52.0/53.5	160.0/160.0	Volleyball	N-Mo	2	8	MB throws, MB unilateral throwing (dominant and non-dominant arm) -1 kg MB-
Pienaar et al. [100]	Yes	NR	18.9/18.9	NR	NR	Rugby	Mo	3	4	Chest pass, step and throw, two-arm put, single arm put, MB crossover push-up, single clap push-up, pullover throws, kneeling side throw, BP throws, plyometric bench press throws, 2 ball MB push-up, MB push ups w/partner, drop and catch push-up, over the head backward throw, and incline push-up with depth jump, lateral shuffle and pass, and quarter-eagle chest pass, medicine ball grab, power drops
Santos et al. [28]	Yes	M	14.7/14.2	72.7/61.1	175.9/173.2	Basketball	N-Mo	2	10	MB chest pass, MB overhead throw, MB backward throw, MB seated chest pass, MB seated, backward throw, MB pullover pass, MB power drop (3 kg MB)
Santos et al. [101]	Yes	M	15.0/14.5	62.6/61.1	172.9/173.2	Basketball	N-Mo	2	10	MB chest pass, MB overhead throw, MB backward throw, MB seated chest pass, MB seated, backward throw, MB pullover pass, MB power drop (3 kg MB)
Schulte-Edelmann et al. [83]	Yes	Mix	NR	NR	NR	NA	NR	2	6	Plyoback device throws and catch (1 kg MB)
Singh Vishen et al. [84]	Yes	M	NR	NR	NR	Cricket	H	3	6	Plyometric push-up
Singla et al. [102]	Yes	M	16.3/19.9/26.9	62.1/70.0/78.1	169.9/171.8/178.1	Cricket	N	3	8	MB 2 kg two-handed overhead throw, MB 3 kg two-handed chest pass, MB 3 kg two-handed side throw, MB 1 kg one-handed baseball throw, MB 1 kg 90/90 external rotation side-throw, MB 1 kg deceleration baseball throw
Sortwell et al. [103]	Yes	Mix	7.3/7.4	NR	NR	NA	L	3	8	MB chest pass, MB throw downs, MB one-arm shoulder pass, MB overhead pass, MB push pass, Explosive push-up on step edge, MB lunge chest pass, Medicine ball push pass, Medicine ball overhead throw downs (1–2 kg MB)
Swanik et al. [85]	Yes	M	20.4/22.0	73.1/74.8	180.0/174.0	NA	N	2	8	Ext rotation ball toss, chest-pass ball toss, overhead-ball toss, plyometric push-up
Szymanski et al. [104]	Yes	M	15.4/15.3	72.5/76.2	176.4/178.4	Baseball	M	3	12	Hitter’s throw, standing Fig. 8, speed rotations, standing side throw, granny throw, standing backwards throw, squat and throw
Thaqi et al. [105]	NR	M	16.0	NR	NR	NA	L	2–4^b^	12	Throws (power drop), plyometric push-up, BP with MB, depth push-up (from box), alternating MB plyometric push-up, push-up (gymnastics parallels)
Turgut et al. [106]	Yes	F	11.0/11.0	45.2/47.1	162.7/159.1	Volleyball	M	3	12	"Ballistic Six" (elastic band shoulder external rotation at 0°, elastic band shoulder external rotation at 90°/90°, overhead throw using a 2 kg medicine ball, 90°/90° external rotation side-throw, deceleration throw, volleyball serve)
Valades et al. [21]	No	F	21.9/27.2	76.0/70.5	183.0/180.0	Volleyball	H	2	8	MB supine reception and chest throw, MB supine reception and overhead throw (30% of 1RM MB)
Vossen et al. [107]	Yes	F	17.3/17.4	56.6/56.9	162.9/165.2	NA	N	3	6	Plyometric push-up
Wilson et al. [108]	Yes	M	20.5/24.5	81.1/75.9	181.0/175.0	RT	N	2	8	MB medicine throws
Young et al. [109]	No	M	19.9	79.1	NR	Mixed	Mo-H	2	5	BP throw

*RM* one repetition maximum, *BP* bench press, *F* female, *Fit* fitness, *Fr* frequency of upper-body plyometric training sessions per week, *H* high, *L* low, *M* male, *MB* medicine ball, *Mo* moderate, *N* normal, *NA* not applicable, *NR* no reported, *Rand* randomized, *RT* resistance training, *BPT* upper-body plyometric training

^a^All values reported as XX.X/XX.X indicate that a range of values was reported in the respective study or that two or more experimental groups were included

^b^The frequency was initially 2 sessions/week, then 3 sessions/week, and then 4 sessions/week

Ten studies [26, 40, 83, 85, 98, 99, 103, 105, 107, 108] examined non-athletes (including resistance-trained participants and physical education students). Other studies examined athletes from different sports such as handball (n = 5) [86, 88, 90, 91, 96], basketball [28, 82, 101], baseball [41, 89, 104], tennis [93, 94, 110], volleyball [20, 21, 106] (n = 3 for each sport), cricket [84, 102] (n = 2), golf [87], karate [97], rugby [100], softball [92], and table tennis [95] (n = 1 for each sport). Of note, one study [109] included participants from different sports (i.e., water polo, field hockey, gymnastics, and volleyball). A total of 1,412 participants, with an age range of 7.3–27.2 years, were analysed in this systematic review. Taking all included studies together, 773 individuals from 52 groups participated in the intervention programs and 639 participated in the control groups (35 groups). Among the 35 control groups, 22 groups were active controls (e.g., handball players), 6 groups were passive controls, and the other 7 groups were intervention control groups (e.g., high-load resistance training). Nineteen experimental groups (and their respective controls) involved participants with a mean age of < 18 years (Table 3). Regarding participants' sex, 6 studies reported a mixed sample of male and females (n = 287 [20.3% of total participants]), 13 groups involved females only (n = 216 [15.3% of total participants]), 44 groups involved males (n = 821 [58.2% of total participants]), and 6 groups involved unspecified participants' sex (n = 88 [6.2% of total participants]) (Table 3). All except six studies [21, 94, 97, 105, 109, 110] recruited experimental and control groups from the same sample through randomization procedures (e.g., groups with similar [probability] training-competition level, age, sport). According to one study [94], pre-tests were employed to regulate the initial status of players. Training duration in the intervention and control groups ranged from 4 to 12 weeks (Table 3), although most studies lasted 8 weeks (54.3%, n = 19). The frequency of weekly training sessions ranged from 2 to 4 sessions per week with a median (and mode) of 2 (Table 3). The testing protocols involved mostly medicine ball throws (n = 17 studies), maximal strength performance (n = 13), and sport-specific throwing performance (n = 10).

### Results From the Meta-Analysis

#### Maximal Strength Performance

**Fig. 2:**
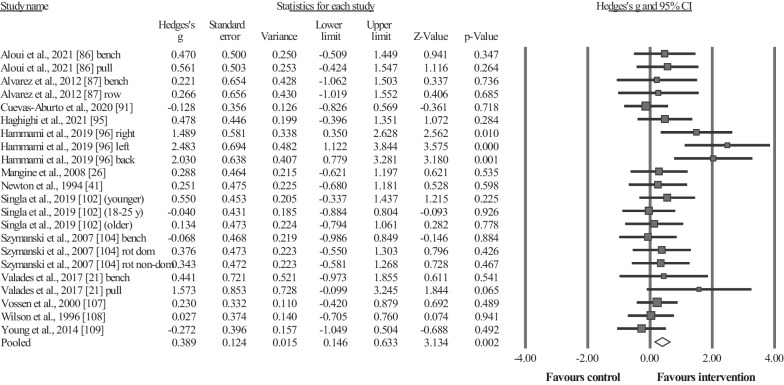
Forest plot illustrating plyometric training-related improvements of the maximal strength performance in comparison to control (i.e., passive, active, specific-active) groups. Forest plot values are shown as effect sizes (Hedges’ g) with 95% confidence intervals (CI). Black squares: individual studies. The size represents the relative weight. White rhomboid: summary value. Rot dom: dominant torso rotational strength; Rot non-dom: non-dominant torso rotational strength

Thirteen studies provided data for maximal strength performance, involving 15 intervention groups and 15 control/comparator groups. Results showed a small significant effect for the intervention groups compared to the control (i.e., passive, active, specific-active) groups: ES = 0.39, 95% CI = 0.15–0.63, *p* = 0.002, *I*^2^ = 29.7%, total participants n = 363 (Fig. 2). Egger's two-tailed test revealed a *p*-value < 0.001, and after the Duval and Tweedie´s trim and fill adjustment method (with three studies trimmed to the right of the mean), the adjusted values indicated an ES = 0.52, 95% CI = 0.27–0.76. After the sensitivity analyses (automated leave-one-out analysis), the robustness of the summary estimates (i.e., *p*-value, ES and 95% CI) was confirmed.

#### Medicine Ball Throwing Performance

**Fig. 3:**
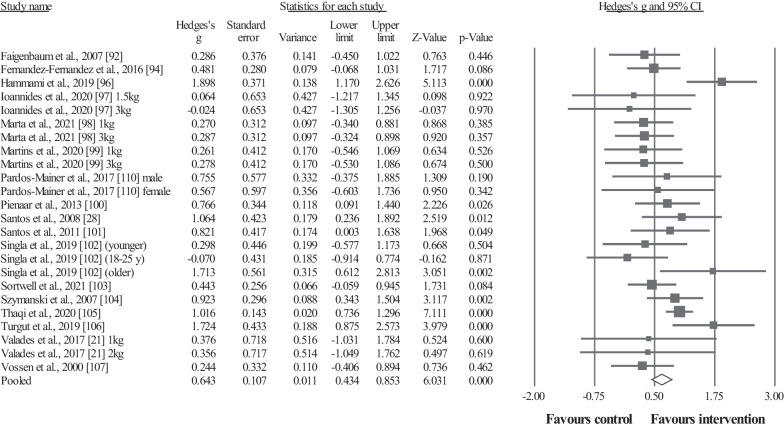
Forest plot illustrating plyometric training-related improvements of the medicine ball throwing performance in comparison to control (i.e., passive, active, specific-active) groups. Forest plot values are shown as effect sizes (Hedges’ g) with 95% confidence intervals (CI). Black squares: individual studies. The size represents the relative weight. White rhomboid: summary value

Seventeen studies provided data for medicine ball throwing (MBT) performance, involving 20 intervention groups and 20 control/comparator groups, using medicine balls with different weights: 1 kg [21, 96, 98, 99, 103], 1.5 kg [97], 2 kg [21, 94, 104, 110], 2.7 kg [107], 3 kg [21, 28, 96–102, 106], 3.6 kg [92], 4 kg [21], and 5 kg [21]. Results showed a significant effect for the intervention groups compared to the control (i.e., passive, active, specific-active) groups: ES = 0.64, 95% CI = 0.43–0.85, *p* < 0.001, *I*^2^ = 46.3%, total participants n = 819, Egger's test two-tailed = 0.229 (Fig. 3). After the sensitivity analyses (automated leave-one-out analysis), the robustness of the summary estimates (i.e., *p*-value, ES and 95% CI) was confirmed.

#### Sport-Specific Throwing Performance

**Fig. 4:**
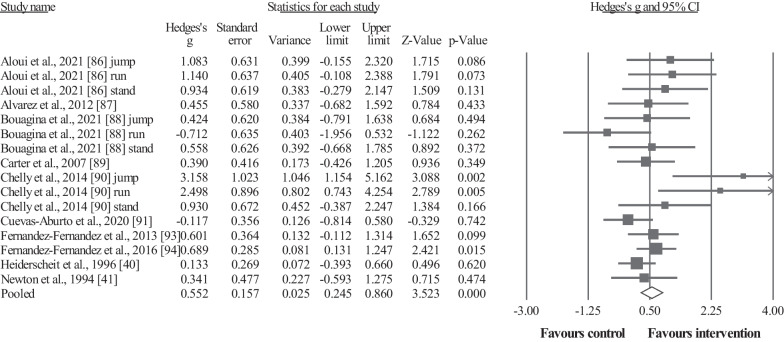
Forest plot illustrating plyometric training-related improvements of the sport-specific throwing performance in comparison to control (i.e., passive, active, specific-active) groups. Forest plot values are shown as effect sizes (Hedges’ g) with 95% confidence intervals (CI). Black squares: individual studies. The size represents the relative weight. White rhomboid: summary value

Ten studies provided data for sport-specific throwing performance, involving 10 intervention groups and 10 control/comparator groups. Results showed a significant effect for the intervention groups compared to the control (i.e., passive, active, specific-active) groups: ES = 0.55, 95% CI = 0.25 to 0.86, *p* < 0.001, *I*^2^ = 36.8%, total participants n = 291 (Fig. 4). Egger's two-tailed test revealed a *p*-value = 0.029, and after the Duval and Tweedie´s trim and fill adjustment method (with five studies trimmed to the left of the mean) the adjusted values indicated an ES = 0.34, 95% CI = − 0.01–0.68. After the sensitivity analyses (automated leave-one-out analysis), the robustness of the summary estimates (i.e., *p*-value, ES and 95% CI) was confirmed.

#### Muscle Volume

**Fig. 5:**
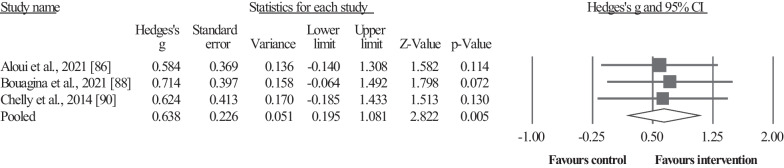
Forest plot illustrating plyometric training-related increase of the upper-body muscle volume in comparison to active controls. Forest plot values are shown as effect sizes (Hedges’ g) with 95% confidence intervals (CI). Black squares: individual studies. The size represents the relative weight. White rhomboid: summary value

Three studies provided data for upper-body muscle volume, involving 3 intervention groups and 3 control/comparator groups. Results showed a significant effect for the intervention groups compared to the active control groups: ES = 0.64, 95% CI = 0.20–1.08, *p* = 0.005, *I*^2^ = 0.0%, total participants n = 78 (Fig. 5).

#### Certainty of Evidence

Results from the GRADE analyses are presented in Table 4. For medicine ball throwing and muscle volume the certainty of evidence was considered low. For maximal strength performance and sport-specific throwing, the evidence was rated as very low.

**Table 4:** GRADE analysis

Outcome	N° studies (sample size)	Risk of bias in studies	Risk of publication bias	Inconsistency	Imprecision	Certainty of evidence
Maximal strength performance	13 (n = 363)	No downgrade (median PEDro score: 6)	Downgrade by 1 level (Egger's two-tailed test, *p* < 0.001)	Downgrade by 1 level (25% < *I*^2^ < 75%)	Downgrade by two levels (n < 800; very large CIs crossing the null effect)	Very low
Medicine ball throwing	17 (n = 819)	No downgrade (median PEDro score: 6)	No downgrade (no suspected risk of publication bias)	Downgrade by 1 level (25% < *I*^2^ < 75%)	Downgrade by one level (very large CIs crossing the null effect)	Low
Sport-specific throwing	10 (n = 291)	No downgrade (median PEDro score: 6)	Downgrade by 1 level (Egger's two-tailed test, *p* = 0.029)	Downgrade by 1 level (25% < *I*^2^ < 75%)	Downgrade by two levels (n < 800; very large CIs crossing the null effect)	Very low
Muscle volume	3 (n = 78)	No downgrade (median PEDro score: 6)	Not applicable (< 10 studies)	No downgrading (*I*^2^ < 25%)	Downgrade by two levels (n < 800; very large CIs crossing the null effect)	Low

Risk of bias in studies: downgraded by one level if the median PEDro scores were moderate (< 6) or by two levels if they were poor (< 4)

Low risk of indirectness was attributed by default due to strict eligibility criteria

Risk of publication bias (assessed only if ≥ 10 studies were available for the comparison): downgraded by one level if there was suspected publication bias

Inconsistency: judgments were downgraded by one or two levels when the impact of statistical heterogeneity (*I*^2^) was moderate (≥ 25%) or high (> 75%)

Imprecision: one level of downgrading occurred whenever < 800 participants were available for a comparison [81] and/or if there was no clear direction of the effects (including large ranges for the 95% confidence interval, even if the overall meta-analysis presents a clear average direction). When both were observed, certainty was downgraded by two levels

*Note*: for outcomes not meta-analytically analysed, a very low certainty of evidence was considered present

#### Adverse Effects

Most of the included studies did not report soreness, pain, fatigue, injury, damage, or adverse health effects related to the UBPT intervention. One study indicated that four participants from the control group were excluded because of unspecified injury and insufficient attendance at training/assessment sessions [88]. Another study reported that 6/30 players from the experimental group and 3/30 from the control group were excluded from the final analysis due to acute injuries (i.e., ankle sprain) produced during plyometric training (only 1 player) or during specific sport training (tennis, 8 players) [94].

## Discussion

The primary goal of this systematic review with meta-analysis was to examine the effects of UBPT on the physical fitness of healthy youth and young adult participants, compared to active, specific-active, and passive matched controls. The main findings indicate that UBPT resulted in small to moderate improvements in maximal strength (ES = 0.52), medicine ball throwing performance (ES = 0.64), sport-specific throwing performance (ES = 0.34), and muscle volume (ES = 0.64) in healthy youth and young adult individuals.

### Maximal Strength Performance

Compared to control conditions (i.e., passive, active, and specific-active), UBPT improved maximal strength performance in healthy individuals with small effect sizes ranging from 0.39 to 0.52. This observed improvement is consistent with several physiological adaptations related to maximal strength performance [111, 112], such as improved neuromuscular function (e.g., power, proprioception, and postural control) [113, 114] and increased muscle activation [115]. Moreover, improvements in muscle–tendon stiffness [116, 117] and architecture [116, 118], including increased muscle cross-sectional area, fibre type distribution, muscle mechanics of the upper and lower limbs (i.e., length. and muscle pennation angle), and neural adaptations such as increased firing rates, increased motoneuron excitability and decreased presynaptic inhibition [119] have also been noted after plyometric training.

Improvements in maximal strength are crucial for success in various sports [7, 120–122], especially those where athletes should overcome larger loads (e.g., throwing events, weightlifting) [123]. However, the percentage of athletes from *maximal strength* sports that incorporate UBPT regularly is relatively low, with ~ 14% in powerlifting and ~ 29% in strongman [124, 125]. Results from this systematic review can be helpful in evidence-based practice, but the limited number of studies available on the effect of UBPT on maximal strength performance precludes a robust analysis of the optimal prescription variables to maximize improvements.

Nevertheless, effective and safe UBPT interventions, with an exercise frequency of 2–3 times per week and lasting between 4 and 12 weeks, can be a valuable addition to resistance training programs aimed at improving maximum strength. The exercises performed during the training sessions mainly consisted of medicine ball throws, push-ups, and bench press throws (at 40–55% 1RM [91, 109]). Although these exercises seem to be effective in improving maximal strength, the magnitude of the improvement was small (ES = 0.39). Future research should explore the use of other exercises that may have a greater impact on maximal strength. In this regard, resistance training performed at ~ 90% of 1RM with maximal volitional concentric contraction velocity has been shown to be a suitable method to focus on the nervous system, and investigations have shown that it is more effective in enhancing force generation than conventional resistance training performed at ~ 70–75% of the 1RM [126]. Therefore, the combination of resistance and plyometric training could be a time-effective method, potentially providing larger improvements in maximal upper-body strength [26, 104, 127]. Nevertheless, it is important to be cautious when interpreting the current results, as the GRADE analysis indicates a very low level of certainty concerning this outcome.

### Medicine Ball Throwing Performance

After examining 17 studies that compared UBPT to various control conditions (i.e., passive, active, specific-active control groups), a moderate increase (ES = 0.64) in MBT performance was observed. Plyometric training can induce physiological adaptations associated with MBT performance, such as enhancement of the elastic properties of the musculotendon unit, neural sequencing optimization and firing rates of the motor units involved [57]. UBPT might also induce improvements in muscular fitness traits associated with MBT, including muscle power, explosiveness (e.g., rate of force development), and coordination [48]. However, the learning effect should be considered, as individuals demonstrated greater improvements in performance with weights similar to those used during training sessions which is in line with the principle of training specificity [97].

When using MBT as a field test in the studies included in this systematic review, variability was observed in testing protocols, including different throwing positions (seated, kneeling, standing), different numbers of maximal throwing trials, and the use of medicine balls with different weights ranging from 1 to 5 kg. Regardless of the testing protocol used, increased MBT performance after UBPT can be an important adaptation for athletes, as it can predict performance improvements in shot put [128] or handball throwing [129, 130].

It is noteworthy that adding plyometric training to resistance training programs was more effective than resistance training alone in improving upper-body power (measured by the seated medicine ball throw) [92], torso and sequential hip-torso-arm rotational strength [104]. However, resistance training has been observed to be more effective than MBT in increasing throwing velocity among individuals without prior experience in resistance training [41]. It appears that the total training workload is the most critical factor in increasing overhead throwing speed [131]. Despite this, the present meta-analysis's findings should be interpreted with caution due to the low certainty of evidence in the GRADE analysis.

### Sport-Specific Throwing Performance

A systematic review and meta-analysis revealed a modest improvement (ES = 0.55) in sport-specific throwing performance after UBPT compared to control groups, including active, specific-active, and passive control groups. UBPT exercises can lead to physiological adaptations that may enhance throwing performance, such as increased firing rates of motor units, improved inter- and intra-muscular coordination, increased muscle fibre contraction velocity, and improvements in force and power generation capabilities [132–135]. Moreover, UBPT exercises predominantly target the velocity components of the force–velocity spectrum, which are important for sport-specific throwing [136]. Other neuromuscular adaptations induced by UBPT exercises may involve a better utilization of the stretch-reflex of the upper-body in a high-velocity ballistic manner, similar to the sport-specific throwing skill [137].

Included studies in this systematic review measured sport-specific throwing performance, including handball throwing velocity (standing, jumping, and three-step running), baseball throwing velocity, and ball speed during golf driving. Previous studies have reported the significant transference effects of plyometric exercises to sport-specific performance in other sports (e.g., soccer, swimming) [29, 31, 32]. Furthermore, UBPT-induced adaptations may lead to improved kinetic characteristics during throwing such as increased force, power, and rate of force development [88]. In addition, the increase in maximal strength (Fig. 2) and MBT performance (Fig. 3) discussed in previous sections may also contribute to sport-specific throwing performance [138]. For instance, a significant association between upper-body maximal strength and ball throwing velocity (r = 0.64–0.69) was previously noted [139, 140]. Results arising from the current meta-analysis should be interpreted with caution as the certainty of evidence is very low for this outcome in the GRADE analysis.

### Upper-Body muscle Volume

The results of this meta-analysis suggest that UBPT increases the upper-body muscle volume compared to the control groups (ES = 0.64). No previous meta-analysis has reported the effects of UBPT on upper-body muscle volume. However, previous meta-analyses have reported plyometric training to be effective in muscle hypertrophy [141] and increased muscle architecture (e.g., muscle thickness, fascicle length, pennation angle) [142] of the lower limbs. Indeed, plyometric exercises have also been reported to increase the calf-girth in physically active adults [143]. Moreover, a systematic review suggested that plyometric training may produce similar effects on whole muscle hypertrophy compared to traditional resistance training methods for lower extremities muscles, at least in the short-term (i.e., 12 weeks) [141]. The physiological basis of such an increment of muscle volume may be attributed to specific muscle fibre recruitment during the plyometric exercises [144]. UBPT is characterized by short duration high-velocity movement that activates the motor units associated with fast-twitch muscle fibres [144] which are suggested to have greater potential for hypertrophy [138, 145]. However, such speculations about hypertrophy response being specific to muscle fibre type have been suggested to be controversial in the literature [138, 146]. Another mechanism that may be responsible for the increase in muscle volume is the increase in the rate of protein synthesis [147], which has the potential to increase the muscle volume. This is noteworthy because body composition can influence physical performance such as speed, change of direction, and upper limb explosive strength [148]. However, results of this meta-analysis should be interpreted with caution due to the low number of studies (n = 3) and the low certainty of evidence obtained in the GRADE analysis.

### Limitations and Directions for Future Research

According to the GRADE analysis, the level of certainty of the evidence for most outcomes ranged from very low to low. Therefore, it is important to exercise caution when interpreting the results of the current meta-analysis. To enhance the reliability and applicability of the findings, future research should aim to overcome these limitations by utilizing larger sample sizes and conducting more randomized controlled trials. It is noteworthy, however, that this study is a significant addition to the field, as it has summarized the existing evidence and pinpointed areas requiring further investigation.

While a significant number of studies were included in this review, UBPT is still relatively understudied compared to lower-body plyometric training. Future studies on UBPT should focus on determining the optimal dose for participants based on their characteristics. Additionally, the extent to which UBPT transfers to sport-specific performance in other upper-body-dominated sports (e.g., badminton, tennis, boxing) remains to be determined. Furthermore, most studies included in this review involved male participants, with only six studies including females (as shown in Table 3). This sex disparity highlights the need for more inclusive and representative studies to fully understand the effects of UBPT on female physical fitness.

### Practical Applications

The findings of this systematic review with meta-analysis have significant implications for coaches and practitioners involved in training programs, aiming to optimize training protocols targeting various aspects of physical fitness and sport-specific performance. Table 5 provides evidence-based practical recommendations for UBPT programming for trainers and practitioners seeking to improve maximal strength, medicine ball and sport-specific throwing performance, and upper-body muscle volume among youth and young adult population.

**Table 5:** Evidence-based practical recommendations for UBPT programming

	Wu	Freq	Dur	Int	Exe	PExe	Sets	Reps	RBS	RBTS^a^	PO
Maximal strength performance (13)^b^	15	2–3	4–12	Max, Mix	1–6	50–100	2–10	3–30	60–240	48–96	V, L, no PO
Medicine ball throwing performance (17)	10–15	2–4	4–12	Max, Mix	1–6	50–100	1–10	4–15	15–240	48–96	V, Freq
Sport-specific throwing performance (10)	3.5–15	2–3	4–8	Max	1–5	50–100	2–6	6–20	30–240	48	V, L, no PO
Upper-body muscle volume (3)	15	2	8	Max	1–3	100	3–5	6–12	240	48	V, L

*Dur* total duration (weeks) of UBPT interventions, *Exe* number of UBPT exercises performed per session, *Freq* frequency of UBPT (sessions per week), *Int* intensity of UBPT intervention exercises; *L* load, *Max* maximal, *PExe* percentage of UBPT exercises in the whole upper-body training programme, *PO* progressive overload (either as intensity, type of exercise, volume, or a combination of these), *Reps* total repetitions performed, *RBS* rest between sets (seconds), *RBTS* rest between UBPT sessions (hours), *UBPT* upper-body plyometric training, *V* volume (repetitions), *Wu* warm-up time (minutes)

^**a**^applicable to training sessions within a given week

^**b**^values in parenthesis denote the number of studies reporting data for the outcome

To maximize the potential benefits derived from UBPT on specific training goals (e.g., maximal strength; medicine ball throwing performance [e.g., maximal power]), practitioners may consider a set of evidence-based practical recommendations indicated in Table 5: (i) include a warm-up (≤ 15 min) before UBPT sessions, (ii) perform 2–4 UBPT sessions per week, (iii) continue for a duration of at least 4 weeks, (iv) include a variety of exercises (e.g., 4–12 per session), particularly for long-term interventions, (v) after adequate technique reached for a particular exercise consider a progression up to maximal-intensity exercises, and (vi) include 1–6 sets of 1–10 repetitions per exercise, for a total of 50–100 repetitions per session.

It should be noted that the practical recommendations presented in this systematic review are based on information from the individual studies, rather than the meta-analyses themselves. Unfortunately, few studies directly compared different variables of UBPT prescription (e.g., exercise type, intensity), which limits our ability to analyse these factors.

## Conclusions

The current systematic review provides evidence to support the effectiveness of UBPT interventions in enhancing physical fitness and performance outcomes in healthy youth and young adults. The findings suggest that UBPT can improve maximal strength, medicine ball throwing performance, sport-specific throwing performance, and muscle volume compared to control groups. However, caution should be exercised when interpreting the results, as the level of certainty of the evidence was found to be between very low and low.

### Supplementary Information

**Additional file 1. Table S1.** Search strategy (code line) for each database.

## Data Availability

The article includes all data generated or analysed during the study, which are presented in the form of tables, figures, and/or Additional file. Any other data requirement can be obtained by making a reasonable request to the corresponding author.

## Abbreviations

1RM: One-repetition maximum
CI: Confidence interval
ES: Effect size
GRADE: Grading of Recommendations Assessment, Development and Evaluation
MBT: Medicine ball throwing
OSF: Open Science Framework platform
PEDro: Physiotherapy Evidence Database
PICOS: Participants, intervention, comparators, outcomes, and study design
PRISMA: Preferred Reporting Items for Systematic reviews and Meta-Analyses
UBPT: Upper-body plyometric training

## References

[1] Stien N, Vereide VA, Saeterbakken AH, Hermans E, Shaw MP, Andersen V (2021). Upper body rate of force development and maximal strength discriminates performance levels in sport climbing. PLoS ONE.

[2] Lijewski M, Burdukiewicz A, Stachoń A, Pietraszewska J (2021). Differences in anthropometric variables and muscle strength in relation to competitive level in male handball players. PLoS ONE.

[3] Sorbie GG, Glen J, Richardson AK (2021). Positive relationships between golf performance variables and upper body power capabilities. J Strength Cond Res.

[4] Lovell DI, Mason D, Delphinus E, McLellan C (2013). Upper and lower body anaerobic performance of semi-elite Rugby League players. J Sports Med Phys Fit.

[5] Nevin J, Smith PM (2021). The relationship between absolute and relative upper-body strength and handcycling performance capabilities. Int J Sports Physiol Perform.

[6] Keiner M, Wirth K, Fuhrmann S, Kunz M, Hartmann H, Haff GG (2021). The influence of upper- and lower-body maximum strength on swim block start, turn, and overall swim performance in sprint swimming. J Strength Cond Res.

[7] Sunde A, Johansen JM, Gjøra M, Paulsen G, Bråten M, Helgerud J (2019). Stronger is better: the impact of upper body strength in double poling performance. Front Physiol.

[8] McKean MR, Burkett BJ (2014). The influence of upper-body strength on flat-water sprint kayak performance in elite athletes. Int J Sports Physiol Perform.

[9] Pickett CW, Nosaka K, Zois J, Hopkins WG, Blazevich AJ (2018). Maximal upper-body strength and oxygen uptake are associated with performance in high-level 200-m sprint kayakers. J Strength Cond Res.

[10] Østerås S, Welde B, Danielsen J, van den Tillaar R, Ettema G, Sandbakk Ø (2016). Contribution of upper-body strength, body composition, and maximal oxygen uptake to predict double poling power and overall performance in female cross-country skiers. J Strength Cond Res.

[11] Fett J, Ulbricht A, Ferrauti A (2020). Impact of physical performance and anthropometric characteristics on serve velocity in elite junior tennis players. J Strength Cond Res.

[12] Fernandez-Fernandez J, Granacher U, Sanz-Rivas D, Sarabia Marín JM, Hernandez-Davo JL, Moya M (2018). Sequencing effects of neuromuscular training on physical fitness in youth elite tennis players. J Strength Cond Res.

[13] Filho JC, Gobbi LT, Gurjão AL, Gonçalves R, Prado AK, Gobbi S (2013). Effect of different rest intervals, between sets, on muscle performance during leg press exercise, in trained older women. J Sports Sci Med.

[14] Laukkanen JA, Voutilainen A, Kurl S, Araujo CGS, Jae SY, Kunutsor SK (2020). Handgrip strength is inversely associated with fatal cardiovascular and all-cause mortality events. Ann Med.

[15] Lu Y, Li G, Ferrari P, Freisling H, Qiao Y, Wu L (2022). Associations of handgrip strength with morbidity and all-cause mortality of cardiometabolic multimorbidity. BMJ (Clinical Research ed).

[16] Kunutsor SK, Voutilainen A, Laukkanen JA (2020). Handgrip strength improves prediction of type 2 diabetes: a prospective cohort study. Ann Med.

[17] Pliner EM, Seo NJ, Ramakrishnan V, Beschorner KE (2019). Effects of upper body strength, hand placement and foot placement on ladder fall severity. Gait Posture.

[18] Toraman A, Yildirim NU (2010). The falling risk and physical fitness in older people. Arch Gerontol Geriatr.

[19] Lin J, Chen T (2012). Diversity of strength training methods: a theoretical approach. Strength Cond J.

[20] Pereira A, Costa AM, Santos P, Figueiredo T, Joao PV (2015). Training strategy of explosive strength in young female volleyball players. Medicina.

[21] Valades Cerrato D, Palao JM, Femia P, Urena A (2018). Effect of eight weeks of upper-body plyometric training during the competitive season on professional female volleyball players. J Sports Med Phys Fit.

[22] Bosco C, Rusko H (1983). The effect of prolonged skeletal muscle stretch-shortening cycle on recoil of elastic energy and on energy expenditure. Acta Physiol Scand.

[23] Turner AN, Jeffreys I (2010). The stretch-shortening cycle: proposed mechanisms and methods for enhancement. Strength Cond.

[24] Ramírez-Campillo R, Andrade DC, Izquierdo M (2013). Effects of plyometric training volume and training surface on explosive strength. J Strength Cond Res.

[25] Markovic G, Simek S, Bradic A (2008). Are acute effects of maximal dynamic contractions on upper-body ballistic performance load specific?. J Strength Cond Res.

[26] Mangine GT, Ratamess NA, Hoffman JR, Faigenbaum AD, Kang J, Chilakos A (2008). The effects of combined ballistic and heavy resistance training on maximal lower- and upper-body strength in recreationally trained men. J Strength Cond Res.

[27] Brandenburg JP (2005). The acute effects of prior dynamic resistance exercise using different loads on subsequent upper-body explosive performance in resistance-trained men. J Strength Cond Res.

[28] Santos EJ, Janeira MA (2008). Effects of complex training on explosive strength in adolescent male basketball players. J Strength Cond Res.

[29] Loturco I, Pereira LA, Kobal R, Zanetti V, Kitamura K, Abad CC (2015). Transference effect of vertical and horizontal plyometrics on sprint performance of high-level U-20 soccer players. J Sports Sci.

[30] Loturco I, Tricoli V, Roschel H, Nakamura FY, Cal Abad CC, Kobal R (2014). Transference of traditional versus complex strength and power training to sprint performance. J Hum Kinet.

[31] Ramirez-Campillo R, Alvarez C, García-Pinillos F, Gentil P, Moran J, Pereira LA (2019). Effects of plyometric training on physical performance of young male soccer players: potential effects of different drop jump heights. Pediatr Exerc Sci.

[32] Ramirez-Campillo R, Perez-Castilla A, Thapa RK, Afonso J, Clemente FM, Colado JC (2022). Effects of plyometric jump training on measures of physical fitness and sport-specific performance of water sports athletes: a systematic review with meta-analysis. Sports Med Open.

[33] Ramirez-Campillo R, Moran J, Chaabene H, Granacher U, Behm DG, García-Hermoso A (2020). Methodological characteristics and future directions for plyometric jump training research: a scoping review update. Scand J Med Sci Sports.

[34] Hayath H, Spargoli G (2016). The immediate effects of different intensities of upper limb plyometric warm-up on bowling speed in cricketers. Preliminary results. Sci Riabil..

[35] Wilcox J, Larson R, Brochu KM, Faigenbaum AD (2006). Acute explosive-force movements enhance bench-press performance in athletic men. Int J Sports Physiol Perform.

[36] Abt G, Boreham C, Davison G, Jackson R, Nevill A, Wallace E (2020). Power, precision, and sample size estimation in sport and exercise science research. J Sports Sci.

[37] Fagard RH, Staessen JA, Thijs L (1996). Advantages and disadvantages of the meta-analysis approach. J Hypertens Suppl.

[38] Murad MH, Asi N, Alsawas M, Alahdab F (2016). New evidence pyramid. Evid Based Med.

[39] Ramirez-Campillo R, Álvarez C, García-Hermoso A, Ramírez-Vélez R, Gentil P, Asadi A (2018). Methodological characteristics and future directions for plyometric jump training research: a scoping review. Sports Med.

[40] Heiderscheit BC, McLean KP, Davies GJ (1996). The effects of isokinetic versus plyometric training on the shoulder internal rotators. J Orthop Sports Phys Ther.

[41] Newton RU, McEvoy KI (1994). Baseball throwing velocity: a comparison of medicine ball training and weight training. J Strength Cond Res.

[42] Singla D, Hussain ME, Moiz JA (2018). Effect of upper body plyometric training on physical performance in healthy individuals: a systematic review. Phys Ther Sport.

[43] Shojania KG, Sampson M, Ansari MT, Ji J, Doucette S, Moher D (2007). How quickly do systematic reviews go out of date? A survival analysis. Ann Intern Med.

[44] Page MJ, McKenzie JE, Bossuyt PM, Boutron I, Hoffmann TC, Mulrow CD (2021). The PRISMA 2020 statement: an updated guideline for reporting systematic reviews. BMJ (Clinical Research ed).

[45] Higgins J, Thomas J, Chandler J, Cumpston M, Li T, Page M, et al. Cochrane Handbook for Systematic Reviews of Interventions version 6.3 (updated February 2022); 2022.

[46] Shea BJ, Reeves BC, Wells G, Thuku M, Hamel C, Moran J (2017). AMSTAR 2: a critical appraisal tool for systematic reviews that include randomised or non-randomised studies of healthcare interventions, or both. BMJ (Clinical Research ed).

[47] Liberati A, Altman DG, Tetzlaff J, Mulrow C, Gøtzsche PC, Ioannidis JPA (2009). The PRISMA statement for reporting systematic reviews and meta-analyses of studies that evaluate healthcare interventions: explanation and elaboration. BMJ (Clinical Research ed).

[48] Stockbrugger BA, Haennel RG (2001). Validity and reliability of a medicine ball explosive power test. J Strength Cond Res.

[49] Garcia-Ramos A, Padial P, Garcia-Ramos M, Conde-Pipo J, Arguelles-Cienfuegos J, Stirn I (2015). Reliability analysis of traditional and ballistic bench press exercises at different loads. J Hum Kinet.

[50] Higgins J, Deeks J. Chapter 7: Selecting studies and collecting data, pp 168–182. In Higgins JPT, Green S (editors) Cochrane Handbook for Systematic Reviews of Interventions Version 510 [updated March 2011] The Cochrane Collaboration, 2011 Available from wwwcochrane-handbookorg. 2011.

[51] Wan X, Wang W, Liu J, Tong T (2014). Estimating the sample mean and standard deviation from the sample size, median, range and/or interquartile range. BMC Med Res Methodol.

[52] Lee DK, In J, Lee S (2015). Standard deviation and standard error of the mean. Korean J Anesthesiol.

[53] Drevon D, Fursa SR, Malcolm AL (2016). Intercoder reliability and validity of WebPlotDigitizer in extracting graphed data. Behav Modif.

[54] de Morton NA (2009). The PEDro scale is a valid measure of the methodological quality of clinical trials: a demographic study. Aust J Physiother.

[55] Maher CG, Sherrington C, Herbert RD, Moseley AM, Elkins M (2003). Reliability of the PEDro scale for rating quality of randomized controlled trials. Phys Ther.

[56] Yamato TP, Maher C, Koes B, Moseley A (2017). The PEDro scale had acceptably high convergent validity, construct validity, and interrater reliability in evaluating methodological quality of pharmaceutical trials. J Clin Epidemiol.

[57] Stojanović E, Ristić V, McMaster DT, Milanović Z (2017). Effect of plyometric training on vertical jump performance in female athletes: a systematic review and meta-analysis. Sports Med.

[58] Asadi A, Arazi H, Young WB, Saez de Villarreal E (2016). The effects of plyometric training on change-of-direction ability: A meta-analysis. Int J Sports Physiol Perform.

[59] Aubut JA, Marshall S, Bayley M, Teasell RW (2013). A comparison of the PEDro and Downs and Black quality assessment tools using the acquired brain injury intervention literature. NeuroRehabilitation.

[60] Moseley AA-O, Rahman P, Wells GA, Zadro JR, Sherrington C, Toupin-April K (2019). Agreement between the Cochrane risk of bias tool and Physiotherapy Evidence Database (PEDro) scale: a meta-epidemiological study of randomized controlled trials of physical therapy interventions. PLoS ONE.

[61] Cashin AG, McAuley JH (2020). Clinimetrics: physiotherapy evidence database (PEDro) scale. J Physiother.

[62] Ramirez-Campillo R, Sanchez-Sanchez J, Romero-Moraleda B, Yanci J, Garcia-Hermoso A, Manuel CF (2020). Effects of plyometric jump training in female soccer player's vertical jump height: a systematic review with meta-analysis. J Sports Sci.

[63] Ramirez-Campillo R, Castillo D, Raya-González J, Moran J, de Villarreal ES, Lloyd RS (2020). Effects of plyometric jump training on jump and sprint performance in young male soccer players: a systematic review and meta-analysis. Sports Med.

[64] Valentine JC, Pigott TD, Rothstein HR (2010). How many studies do you need?: a primer on statistical power for meta-analysis. J Ed Behavioral Stat..

[65] García-Hermoso A, Ramírez-Campillo R, Izquierdo M (2019). Is muscular fitness associated with future health benefits in children and adolescents? a systematic review and meta-analysis of longitudinal studies. Sports Med.

[66] Moran J, Ramirez-Campillo R, Granacher U (2018). Effects of jumping exercise on muscular power in older adults: a meta-analysis. Sports Med.

[67] Jackson D, Turner R (2017). Power analysis for random-effects meta-analysis. Res Synth Methods.

[68] Deeks JJ, Higgins JP, Altman DG. Analysing data and undertaking meta-analyses. In: Higgins JP, Green S, editors. Cochrane handbook for systematic reviews of interventions: the Cochrane collaboration; 2008. p. 243–96.

[69] Kontopantelis E, Springate DA, Reeves D (2013). A re-analysis of the Cochrane Library data: the dangers of unobserved heterogeneity in meta-analyses. PLoS ONE.

[70] Hopkins WG, Marshall SW, Batterham AM, Hanin J (2009). Progressive statistics for studies in sports medicine and exercise science. Med Sci Sports Exerc.

[71] Higgins JP, Deeks JJ, Altman DG, Higgins JP, Green S (2008). Special topics in statistics. Cochrane handbook for systematic reviews of interventions: The Cochrane Collaboration.

[72] Higgins JP, Thompson SG (2002). Quantifying heterogeneity in a meta-analysis. Stat Med.

[73] Higgins, Thomas J, Chandler J, Cumpston M, Li T, Page MJ, et al. Assessing risk of bias due to missing results in a synthesis. In: Cochrane handbook for systematic reviews of interventions. Second edition. pp 365 Chichester (UK): John Wiley & Sons; 2019.

[74] Sterne JAC, Sutton AJ, Ioannidis JPA, Terrin N, Jones DR, Lau J (2011). Recommendations for examining and interpreting funnel plot asymmetry in meta-analyses of randomised controlled trials. BMJ (Clinical Research ed).

[75] Egger M, Davey Smith G, Schneider M, Minder C (1997). Bias in meta-analysis detected by a simple, graphical test. BMJ (Clinical Research Ed).

[76] Duval S, Tweedie R (2000). Trim and fill: a simple funnel-plot-based method of testing and adjusting for publication bias in meta-analysis. Biometrics.

[77] Shi L, Lin L (2019). The trim-and-fill method for publication bias: practical guidelines and recommendations based on a large database of meta-analyses. Medicine.

[78] Guyatt GH, Oxman AD, Akl EA, Kunz R, Vist G, Brozek J (2011). GRADE guidelines: 1. Introduction-GRADE evidence profiles and summary of findings tables. J Clin Epidemiol.

[79] Zhang Y, Alonso-Coello P, Guyatt GH, Yepes-Nuñez JJ, Akl EA, Hazlewood G (2019). GRADE Guidelines: 19. Assessing the certainty of evidence in the importance of outcomes or values and preferences—Risk of bias and indirectness. J Clin Epidemiol.

[80] Zhang Y, Coello PA, Guyatt GH, Yepes-Nuñez JJ, Akl EA, Hazlewood G (2019). GRADE guidelines: 20. Assessing the certainty of evidence in the importance of outcomes or values and preferences—inconsistency, imprecision, and other domains. J Clin Epidemiol.

[81] Guyatt G, Oxman AD, Kunz R, Brozek J, Alonso-Coello P, Rind D, et al. Corrigendum to GRADE guidelines 6. Rating the quality of evidence-imprecision. J Clin Epidemiol 2011;64:1283–1293. Journal of clinical epidemiology. 2021;137:265. 10.1016/j.jclinepi.2021.04.014 34174652

[82] Hasan N, Nuhmani S, Kachanathu SJ, Muaidi QI (2018). Efficacy of complex training on angular velocity of shoulder in collegiate basketball players. J Back Musculoskelet Rehabil.

[83] Schulte-Edelmann JA, Davies GJ, Kernozek TW, Gerberding ED (2005). The effects of plyometric training of the posterior shoulder and elbow. J Strength Cond Res.

[84] Singh Vishen PK, Sen S (2015). Comparision of dynamic push- up training and plyometric push-up training on upper body performance test in cricket player. Int J Phys Educ Sports Health.

[85] Swanik KA, Thomas SJ, Struminger AH, Bliven KC, Kelly JD, Swanik CB (2016). The effect of shoulder plyometric training on amortization time and upper-extremity kinematics. J Sport Rehabil.

[86] Aloui G, Hermassi S, Hammami M, Cherni Y, Gaamouri N, Shephard RJ (2021). Effects of elastic band based plyometric exercise on explosive muscular performance and change of direction abilities of male team handball players. Front Physiol.

[87] Alvarez M, Sedano S, Cuadrado G, Redondo JC (2012). Effects of an 18-week strength training program on low-handicap golfers' performance. J Strength Cond Res.

[88] Bouagina R, Padulo J, Fray A, Larion A, Abidi H, Chtara M (2022). Short-term in-season ballistic training improves power, muscle volume and throwing velocity in junior handball players. A randomized control trial. Biol Sport.

[89] Carter AB, Kaminski TW, Douex AT, Knight CA, Richards JG (2007). Effects of high volume upper extremity plyometric training on throwing velocity and functional strength ratios of the shoulder rotators in collegiate baseball players. J Strength Cond Res.

[90] Chelly MS, Hermassi S, Aouadi R, Shephard RJ (2014). Effects of 8-week in-season plyometric training on upper and lower limb performance of elite adolescent handball players. J Strength Cond Res.

[91] Cuevas-Aburto J, Janicijevic D, Perez-Castilla A, Chirosa-Rios LJ, Garcia-Ramos A (2020). Changes in bench press performance and throwing velocity after strength-oriented and ballistic resistance training programs. J Sports Med Phys Fit.

[92] Faigenbaum AD, McFarland JE, Keiper FB, Tevlin W, Ratamess NA, Kang J (2007). Effects of a short-term plyometric and resistance training program on fitness performance in boys age 12 to 15 years. J Sports Sci Med.

[93] Fernandez-Fernandez J, Ellenbecker T, Sanz-Rivas D, Ulbricht A, Ferrautia A (2013). Effects of a 6-week junior tennis conditioning program on service velocity. J Sports Sci Med.

[94] Fernandez-Fernandez J, Saez de Villarreal E, Sanz-Rivas D, Moya M (2016). The effects of 8-week plyometric training on physical performance in young tennis players. Pediatr Exerc Sci.

[95] Haghighi AH, Zaferanieh A, Hosseini-Kakhak SA, Maleki A, Esposito F, Ce E (2021). Effects of power and ballistic training on table tennis players' electromyography changes. Int J Environ Res Public Health.

[96] Hammami M, Ramirez-Campillo R, Gaamouri N, Aloui G, Shephard RJ, Chelly MS (2019). Effects of a combined upper- and lower-limb plyometric training program on high-intensity actions in female U14 handball players. Pediatr Exerc Sci.

[97] Ioannides C, Apostolidis A, Hadjicharalambous M, Zaras N (2020). Effect of a 6-week plyometric training on power, muscle strength, and rate of force development in young competitive karate athletes. J Phys Educ Sport.

[98] Marta C, Alves AR, Casanova N, Neiva HP, Marinho DA, Izquierdo M (2022). Suspension versus plyometric training in children's explosive strength. J Strength Cond Res.

[99] Martins J, Cardoso J, Honorio S, Silva A (2020). The effect of a strength training programme in adolescents in physical education classes. Retos.

[100] Pienaar C, Coetzee B (2013). Changes in selected physical, motor performance and anthropometric components of university-level rugby players after one microcycle of a combined rugby conditioning and plyometric training program. J Strength Cond Res.

[101] Santos EJAM, Janeira MAAS (2011). The effects of plyometric training followed by detraining and reduced training periods on explosive strength in adolescent male basketball players. J Strength Cond Res.

[102] Singla D, Hussain ME (2019). Adaptations of the upper body to plyometric training in cricket players of different age groups. J Sport Rehabil.

[103] Sortwell A, Newton M, Marinho DA, Ferraz R, Perlman D (2021). The effects of an eight week plyometric-based program on motor performance skills and muscular power in 7–8-year-old primary school students. Int J Kinesiol Sports Sci.

[104] Szymanski DJ, Szymanski JM, Bradford TJ, Schade EL, Pascoe DD (2007). Effect of twelve weeks of medicine ball training on high school baseball players. J Strength Cond Res.

[105] Thaqi A, Berisha M, Hoxha S (2020). The effect of plyometric training on the power-related factors of children aged 16 years-old. Prog Nutr.

[106] Turgut E, Cinar-Medeni O, Colakoglu FF, Baltaci G (2019). "Ballistic Six" upper-extremity plyometric training for the pediatric volleyball players. J Strength Cond Res.

[107] Vossen JF, Kramer JE, Burke DG, Vossen DP (2000). Comparison of dynamic push-up training and plyometric push-up training on upper-body power and strength. J Strength Cond Res.

[108] Wilson GJ, Murphy AJ, Giorgi A (1996). Weight and plyometric training: effects on eccentric and concentric force production. Can J Appl Physiol.

[109] Young KP, Haff GG, Newton RU, Gabbett TJ, Sheppard JM (2015). Assessment and monitoring of ballistic and maximal upper-body strength qualities in athletes. Int J Sports Physiol Perform.

[110] Pardos-Mainer E, Ustero-Pérez O, Gonzalo-Skok O (2017). Effects of upper and lower body plyometric training on physical performance in young tennis players. RICYDE Rev Int Cienc Deporte.

[111] Trezise J, Blazevich AJ (2019). Anatomical and neuromuscular determinants of strength change in previously untrained men following heavy strength training. Front Physiol.

[112] Suchomel TJ, Nimphius S, Bellon CR, Stone MH (2018). The importance of muscular strength: training considerations. Sports Med.

[113] Myer GD, Ford KR, Brent JL, Hewett TE (2006). The effects of plyometric versus dynamic stabilization and balance training on power, balance, and landing force in female athletes. J Strength Cond Res.

[114] Swanik KA, Lephart SM, Swanik CB, Lephart SP, Stone DA, Fu FH (2002). The effects of shoulder plyometric training on proprioception and selected muscle performance characteristics. J Shoulder Elbow Surg.

[115] Lephart SM, Abt JP, Ferris CM, Sell TC, Nagai T, Myers JB (2005). Neuromuscular and biomechanical characteristic changes in high school athletes: a plyometric versus basic resistance program. Br J Sports Med.

[116] Kubo K, Ishigaki T, Ikebukuro T (2017). Effects of plyometric and isometric training on muscle and tendon stiffness in vivo. Physiol Rep.

[117] Hirayama K, Iwanuma S, Ikeda N, Yoshikawa A, Ema R, Kawakami Y (2017). Plyometric training favors optimizing muscle-tendon behavior during depth jumping. Front Physiol.

[118] Kubo K, Morimoto M, Komuro T, Yata H, Tsunoda N, Kanehisa H (2007). Effects of plyometric and weight training on muscle-tendon complex and jump performance. Med Sci Sports Exerc.

[119] Aagaard P (2003). Training-induced changes in neural function. Exerc Sport Sci Rev.

[120] Bragazzi NL, Rouissi M, Hermassi S, Chamari K (2020). Resistance training and handball players' isokinetic, isometric and maximal strength, muscle power and throwing ball velocity: a systematic review and meta-analysis. Int J Environ Res Public Health.

[121] Ebben WP, Hintz MJ, Simenz CJ (2005). Strength and conditioning practices of Major League Baseball strength and conditioning coaches. J Strength Cond Res.

[122] Torres-Ronda L, Delextrat A, Gonzalez-Badillo JJ (2014). The relationship between golf performance, anthropometrics, muscular strength and power characteristics in young elite players : original research article. Int J Sports Med.

[123] Stone MH, Moir G, Glaister M, Sanders R (2002). How much strength is necessary?. Phys Ther Sport.

[124] Winwood PW, Keogh JW, Harris NK (2011). The strength and conditioning practices of strongman competitors. J Strength Cond Res.

[125] Swinton PA, Lloyd R, Agouris I, Stewart A (2009). Contemporary training practices in elite British powerlifters: survey results from an international competition. J Strength Cond Res.

[126] Heggelund J, Fimland MS, Helgerud J, Hoff J (2013). Maximal strength training improves work economy, rate of force development and maximal strength more than conventional strength training. Eur J Appl Physiol.

[127] Fathi A, Hammami R, Moran J, Borji R, Sahli S, Rebai H (2019). Effect of a 16-week combined strength and plyometric training program followed by a detraining period on athletic performance in pubertal volleyball players. J Strength Cond Res.

[128] Zaras N, Stasinaki A, Arnaoutis G, Terzis G (2016). Predicting throwing performance with field tests. New Stud Athl.

[129] Fathloun M, Hermassi S, Chelly MS, Bensbaa A (2011). Relationship between medicine ball explosive power tests, throwing ball velocity and jump performance in team handball players. Pedagog Psychol Med Biol Probl Phys Train Sports.

[130] Raeder C, Fernandez-Fernandez J, Ferrauti A (2015). Effects of six weeks of medicine ball training on throwing velocity, throwing precision, and isokinetic strength of shoulder rotators in female handball players. J Strength Cond Res.

[131] van den Tillaar R, Marques MC (2013). Effect of different training workload on overhead throwing performance with different weighted balls. J Strength Cond Res.

[132] Malisoux L, Francaux M, Nielens H, Renard P, Lebacq J, Theisen D (2006). Calcium sensitivity of human single muscle fibers following plyometric training. Med Sci Sports Exerc.

[133] Malisoux L, Francaux M, Nielens H, Theisen D (2006). Stretch-shortening cycle exercises: an effective training paradigm to enhance power output of human single muscle fibers. J Appl Physiol.

[134] Malisoux L, Francaux M, Theisen D (2005). Effect of plyometric training on mechanical properties of human single muscle fibres: 1520 10:30 AM–10:45 AM. Med Sci Sports Exerc.

[135] Markovic G, Mikulic P (2010). Neuro-musculoskeletal and performance adaptations to lower-extremity plyometric training. Sports Med.

[136] Turner AN, Comfort P, McMahon J, Bishop C, Chavda S, Read P (2020). Developing powerful athletes, Part 1: mechanical underpinnings. Strength Cond.

[137] Cormie P, McGuigan MR, Newton RU (2011). Developing maximal neuromuscular power: Part 1–biological basis of maximal power production. Sports Med.

[138] Ogborn D, Schoenfeld BJ (2014). The role of fiber types in muscle hypertrophy: implications for loading strategies. Strength Cond.

[139] Marques MC, van den Tilaar R, Vescovi JD, Gonzalez-Badillo JJ (2007). Relationship between throwing velocity, muscle power, and bar velocity during bench press in elite handball players. Int J Sports Physiol Perform.

[140] Chelly MS, Hermassi S, Shephard RJ (2010). Relationships between power and strength of the upper and lower limb muscles and throwing velocity in male handball players. J Strength Cond Res.

[141] Grgic J, Schoenfeld BJ, Mikulic P (2021). Effects of plyometric vs resistance training on skeletal muscle hypertrophy: A review. J Sport Health Sci.

[142] Ramírez-delaCruz M, Bravo-Sánchez A, Esteban-García P, Jiménez F, Abián-Vicén J (2022). Effects of plyometric training on lower body muscle architecture, tendon structure, stiffness and physical performance: a systematic review and meta-analysis. Sports Med Open.

[143] Singh G, Kushwah GS, Singh T, Thapa RK, Granacher U, Ramirez-Campillo R (2022). Effects of sand-based plyometric-jump training in combination with endurance running on outdoor or treadmill surface on physical fitness in young adult males. J Sports Sci Med.

[144] Macaluso F, Isaacs AW, Myburgh KH (2012). Preferential type II muscle fiber damage from plyometric exercise. J Athl Train.

[145] Folland JP, Williams AG (2007). The adaptations to strength training: morphological and neurological contributions to increased strength. Sports Med.

[146] Schoenfeld BJ, Vigotsky AD, Grgic J, Haun C, Contreras B, Delcastillo K (2020). Do the anatomical and physiological properties of a muscle determine its adaptive response to different loading protocols?. Physiol Rep.

[147] Watt PW, Kelly FJ, Goldspink DF, Goldspink G (1982). Exercise-induced morphological and biochemical changes in skeletal muscles of the rat. J Appl Physiol Respir Environ Exerc Physiol.

[148] Rinaldo N, Toselli S, Gualdi-Russo E, Zedda N, Zaccagni L (2020). Effects of anthropometric growth and basketball experience on physical performance in pre-adolescent male players. Int J Environ Res Public Health.

