# Supplementary material for: Effects of Upper-Body Plyometric Training on Physical Fitness in Healthy Youth and Young Adult Participants: A Systematic Review with Meta-Analysis
Source: Sports Med Open. 2023 Oct 13;9:93. doi: 10.1186/s40798-023-00631-2 (PMC10575843; doi:10.1186/s40798-023-00631-2)
Supplement: Supplementary file 1 — Additional file 1. Table S1. Search strategy (code line) for each database. [file 40798_2023_631_MOESM1_ESM.docx]

**Electronic Supplementary Material Table S1**

**Article title**: Effects of upper-body plyometric training on physical fitness in healthy youth and young adult participants: A systematic review with meta-analysis

**Author names**: Exal Garcia-Carrillo, Rodrigo Ramirez-Campillo, Rohit K. Thapa, José Afonso, Urs Granacher, Mikel Izquierdo

**Affiliation and e-mail of the corresponding author**:

Mikel Izquierdo, PhD

Department of Health Sciences, Public University of Navarra, Av. De Barañain s/n 31008 Pamplona (Navarra) SPAIN. Email: mikel.izquierdo@unavarra.es

Table S1. Search strategy (code line) for each database.

| **Databases** | PubMed | WOS (Core Collection) | Scopus |
| --- | --- | --- | --- |
| **Keywords** | “plyometric”, “ballistic”, “explosive”, “upper body”, “upper limb” | “plyometric”, “ballistic”, “explosive”, “upper body”, “upper limb” | “plyometric”, “ballistic”, “explosive”, “upper body”, “upper limb” |
| **Database fields for the search** | All | All | title, abstract, keywords |
| **Restrictions for the search** | None | None | None |
| **Examples of search strategy code line** | (plyometric) AND (upper body)  (ballistic) AND (upper body)  (explosive) AND (upper body)  (explosive[Title/Abstract]) AND (upper body[Title/Abstract])  (plyometric) AND (upper limb)  (ballistic) AND (upper limb)  (explosive) AND (upper limb)  (explosive[Title/Abstract]) AND (upper limb[Title/Abstract])  (upper body) OR (upper limb) AND (plyometric)  (upper body) OR (upper limb) OR (ballistic) AND (plyometric)  (upper*) OR (ballistic) AND (plyometric)  (upper*) OR (ballistic) OR (explosive) AND (plyometric)  (explosive[Title/Abstract]) OR (plyometric*[Title/Abstract]) OR (ballistic*[Title/Abstract]) AND (upper limb[Title/Abstract]) | (plyometric) AND (upper body)  (ballistic) AND (upper body)  (explosive) AND (upper body)  (TS=(explosive)) AND TS=(upper body)  (plyometric) AND (upper limb)  (ballistic) AND (upper limb)  (explosive) AND (upper limb)  (TS=(explosive)) AND TS=(upper limb) | (plyometric) AND (upper body)  (ballistic) AND (upper body)  (explosive) AND (upper body)  TITLE-ABS-KEY ( explosive AND upper body )  (plyometric) AND (upper limb)  (ballistic) AND (upper limb)  (explosive) AND (upper limb)  TITLE-ABS-KEY ( explosive AND upper limb )  (upper body) OR (upper limb) AND (plyometric)  (upper body) OR (upper limb) OR (ballistic) AND (plyometric)  (upper*) OR (ballistic) AND (plyometric)  (upper*) OR (ballistic) OR (explosive) AND (plyometric)  explosive OR plyometric* OR ballistic* AND upper limb (topic)  explosive OR plyometric* OR ballistic* AND upper limb (topic) |

**Electronic Supplementary Material Table S2**

**Article title**: Effects of upper-body plyometric training on physical fitness in healthy youth and young adult participants: A systematic review with meta-analysis

**Author names**: Exal Garcia-Carrillo, Rodrigo Ramirez-Campillo, Rohit K. Thapa, José Afonso, Urs Granacher, Mikel Izquierdo

**Affiliation and e-mail of the corresponding author**:

Mikel Izquierdo, PhD

Department of Health Sciences, Public University of Navarra, Av. De Barañain s/n 31008 Pamplona (Navarra) SPAIN. Email: mikel.izquierdo@unavarra.es

Table S2. Additional exclusion criteria.

| Excluded were books, book chapters, and congress abstracts, as well as cross-sectional and review papers, and training-related studies that did not focus on the effects of upper body plyometric training (UBPT) exercises, such as plyometric training without the use of upper body (e.g., plyometric jumps only). Also excluded were retrospective studies, prospective studies, studies in which the use of UBPT exercises was not clearly described (e.g., authors stated “plyometric exercises were implemented”, without further explanation), studies for which only the abstract was available, case reports, special communications, letters to the editor, invited commentaries, errata, studies with doubtful quality or unclear peer-review process from the journal [1], overtraining studies, and detraining studies. In the case of detraining studies, these were considered for inclusion if involved a training period prior to a detraining period. |
| --- |

1. Grudniewicz A, Moher D, Cobey KD, Bryson GL, Cukier S, Allen K, et al. Predatory journals: no definition, no defence. Nature. 2019 Dec;576(7786):210-2.
